# Prevalence and characteristics of chronic kidney disease in the Hamburg City Health Study

**DOI:** 10.1093/ndt/gfaf075

**Published:** 2025-04-30

**Authors:** Christian Schmidt-Lauber, Christina Thompson, Elisa Alba Schmidt, Alexandre Klopp, Ammar Alabdo, Tanja Zeller, Ines Schäfer, Raphael Twerenbold, Christoffer Johansen, Stefan Blankenberg, Tobias B Huber

**Affiliations:** III. Department of Medicine, University Medical Center Hamburg-Eppendorf, Hamburg, Germany; Hamburg Center for Kidney Health (HCKH), University Medical Center Hamburg-Eppendorf, Hamburg, Germany; III. Department of Medicine, University Medical Center Hamburg-Eppendorf, Hamburg, Germany; Hamburg Center for Kidney Health (HCKH), University Medical Center Hamburg-Eppendorf, Hamburg, Germany; III. Department of Medicine, University Medical Center Hamburg-Eppendorf, Hamburg, Germany; Hamburg Center for Kidney Health (HCKH), University Medical Center Hamburg-Eppendorf, Hamburg, Germany; III. Department of Medicine, University Medical Center Hamburg-Eppendorf, Hamburg, Germany; Hamburg Center for Kidney Health (HCKH), University Medical Center Hamburg-Eppendorf, Hamburg, Germany; III. Department of Medicine, University Medical Center Hamburg-Eppendorf, Hamburg, Germany; Hamburg Center for Kidney Health (HCKH), University Medical Center Hamburg-Eppendorf, Hamburg, Germany; Department of Cardiology, University Heart and Vascular Center Hamburg-Eppendorf, University Medical Center Hamburg-Eppendorf, Hamburg, Germany; Department of Cardiology, University Heart and Vascular Center Hamburg-Eppendorf, University Medical Center Hamburg-Eppendorf, Hamburg, Germany; Department of Cardiology, University Heart and Vascular Center Hamburg-Eppendorf, University Medical Center Hamburg-Eppendorf, Hamburg, Germany; German Center for Cardiovascular Research (DZHK) Partner Site Hamburg-Kiel-Lübeck, Hamburg, Germany; Institute of Clinical Medicine, Faculty of Health, Copenhagen University and Center for Cancer Late Effect Research CASTLE, Department of Oncology, Rigshospitalet, Copenhagen, Denmark; Department of Cardiology, University Heart and Vascular Center Hamburg-Eppendorf, University Medical Center Hamburg-Eppendorf, Hamburg, Germany; German Center for Cardiovascular Research (DZHK) Partner Site Hamburg-Kiel-Lübeck, Hamburg, Germany; III. Department of Medicine, University Medical Center Hamburg-Eppendorf, Hamburg, Germany; Hamburg Center for Kidney Health (HCKH), University Medical Center Hamburg-Eppendorf, Hamburg, Germany

To the Editor,

The impact of chronic kidney disease (CKD) on worldwide morbidity and mortality is constantly on the rise, underscoring its growing challenge for patients and caregivers. Within recent decades, CKD has emerged as the eighth leading cause of disability-adjusted life years in adults [1]. However, the prevalence and characteristics of CKD vary across populations and current data for Germany remain scarce [2]. This study aims to investigate the CKD prevalence and characteristics in the Hamburg City Health Study (HCHS), considering different kidney function measurements and estimates. The HCHS is an ongoing and prospective population-based cohort study aiming to investigate prognostic factors for major chronic diseases [3]. It encompasses randomly selected inhabitants of the city of Hamburg, Germany, aged 45–74 years. A detailed description of the methods can be found in the Supplementary data, Item S1.

Baseline characteristics are outlined in Table 1. Median age was 63 years [interquartile range (IQR) 55; 70] and 51% were female. Median estimated glomerular filtration rate (eGFR), according to the 2009 CKD-Epidemiology Collaboration (CKD-EPI) formula for creatinine, and urinary albumin-to-creatinine ratio (uACR) were 86 (IQR 75; 94) mL/min/1.73 m^2^ and 5 (IQR 3; 9) mg/g, respectively (Supplementary data, Fig. S1). CKD prevalence, defined as an eGFR <60 mL/min/1.73 m² or uACR ≥30 mg/g, was 11.2% [95% confidence interval (CI) 10.6–11.8%, 1115 individuals]. Six percent (95% CI 5.6%–6.5%, 599 individuals) had an eGFR <60 mL/min/1.73 m² and 6.3% (95% CI 5.9%–6.8%, 632 individuals) an uACR ≥30 mg/g (Fig. 1). CKD prevalence varied widely depending on the formula used for eGFR calculation (Supplementary data, Table S1). The 2012 CKD-EPI formula for cystatin C revealed a prevalence of 19.5% (95% CI 18.7%–20.3%) and the combined formula for creatinine and cystatin C a prevalence of 13.2% (95% CI 12.5%–13.9%, Supplementary data, Table S1). Dipstick proteinuria had moderate sensitivity in predicting uACR (Supplementary data, Table S2).

**Figure 1:**
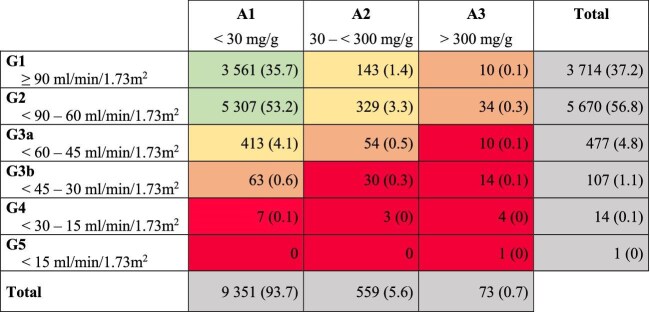
Prevalence of CKD stages in the HCHS. Numbers are counts with percentages in brackets. Colors highlight the risk of CKD progression with low (green), moderate (yellow), high (orange) and very high risk (red).

**Table 1:** Baseline characteristics.

Characteristics	Overall (*n* = 9983)	eGFR <60 mL/min/1.73 m² (*n* = 599)	uACR ≥30 mg/g (*n* = 632)
Demographics			
Age in years, median (IQR)	63 (55; 70)	71 (66; 74)	68 (61; 72)
Female sex, *n* (%)	5 099 (51)	267 (45)	242 (38)
BMI in kg/m², median (IQR)	26.2 (23.7; 29.1)	28.1 (25.3; 31.5)	27.8 (24.6; 31.1)
Heart rate in bpm, median (IQR)	69 (63; 76)	69 (62; 77)	72 (64; 80)
Systolic blood pressure, median (IQR)	138 (126; 151)	142 (129; 155)	147 (132; 162)
Diastolic blood pressure, median (IQR)	82 (76; 88)	81 (74; 87)	83 (76; 92)
Ethnicity, *n* (%)			
Caucasian	9 816 (98)	594 (99)	612 (97)
Black	30 (0)	1 (0)	8 (1)
Other	137 (1)	4 (1)	12 (2)
Comorbidities, *n* (%)			
Hypertension	6 465 (65)	530 (89)	537 (85)
Dyslipidemia	2 273 (23)	261 (44)	228 (36)
Malignancies	1 603 (16)	166 (28)	132 (21)
Diabetes	804 (8)	134 (22)	157 (25)
Coronary heart disease	475 (5)	78 (13)	61 (10)
Heart failure	332 (3)	67 (11)	64 (10)
Peripheral artery disease	323 (3)	45 (8)	40 (6)
Socioeconomics, *n* (%)			
Employment			
Full-time	3 545 (36)	67 (11)	135 (21)
Part-time	1 303 (13)	36 (6)	60 (10)
Unemployed	5 135 (51)	496 (83)	437 (69)
Education			
High	4 455 (45)	224 (37)	250 (40)
Medium	5 063 (51)	332 (55)	349 (55)
Low	465 (5)	43 (7)	33 (5)
Lifestyle			
Smoking status, *n* (%)			
Current	1 980 (20)	90 (15)	157 (25)
Former	4 429 (44)	301 (50)	290 (46)
Never	3 574 (36)	208 (35)	185 (29)
Physical activity in hours per week, median (IQR)	2 (0; 4)	2 (0; 3)	2 (0; 3)
Medication, *n* (%)			
Antihypertensive medication	3 279 (33)	380 (63)	351 (56)
ACE-I or ARB	2 930 (29)	321 (54)	294 (47)
Lipid-lowering medication	1 737 (17)	231 (39)	190 (30)
MRA	69 (1)	26 (4)	8 (1)
SGLT2-I	18 (0)	2 (0)	3 (1)
Laboratory results, median (IQR)			
Hemoglobin in g/dL	14.3 (13.6; 15.1)	14.1 (13.3; 15)	14.5 (13.6; 15.3)
HBA1c in %	5.5 (5.3; 5.8)	5.7 (5.4; 6.1)	5.7 (5.4; 6.2)
Sodium in mmol/L	139 (138, 141)	139 (138; 141)	139 (137; 140)
Potassium in mmol/L	3.9 (3.7; 4)	4.0 (3.8; 4.2)	3.9 (3.7; 4.1)
Creatinine in mg/dL	0.8 (0.7; 1)	1.3 (1.1; 1.4)	0.9 (0.8; 1.1)
Cystatin C in mg/L	1 (0.9; 1.1)	1.4 (1.2; 1.5)	1.1 (1; 1.3)
uACR in mg/g	5 (3; 9)	7 (3; 19)	65 (42; 138)
uPCR in mg/g	88 (65; 143)	102 (70; 182)	211 (146; 341)

Percentages do not sum up to 100% because of rounding.

ACE-I, angiotensin-converting enzyme inhibitors; ARB, angiotensin II receptor blocker; BMI, body mass index; HBA1c, hemoglobin A1c; MRA, mineralocorticoid receptor antagonist; SGLT2-I, sodium glucose transporter II inhibitor; uPCR, urinary protein-to-creatinine ratio.

Although median eGFR and uACR were similar between females and males, the prevalence of an eGFR <60 mL/min/1.73 m² (6.8%, 95% CI 6.1%–7.5% versus 5.2%, 95% CI 4.7%–5.9%) and uACR ≥ 30 mg/g (8%, 95% CI 7.3%–8.8% versus 4.7%, 95% CI 4.2%–5.4%) was higher in males (Supplementary data, Table S3). Consequently, males had a higher CKD prevalence as compared with females (13.1%, 95% CI 12.2%–14.1% versus 9.3%, 95% CI 8.6%–10.1%). The prevalence among individuals with diabetes was highest, at 30.2% (95% CI 27.2%–33.5%). Multivariable logistic regression models showed that age, body mass index, physical activity, hypertension, diabetes and dyslipidemia were the major independent CKD risk factors (Supplementary data, Table S4). Male sex was also associated with CKD, although the association significantly diminished after adjustment for potential confounders, which were more prevalent in males (Supplementary data, Table S5).

This study shows that the current CKD prevalence in a large German population-based cohort is 11.2%, underscoring the importance of CKD in general health. The numbers correspond to other developed countries including the USA, Italy and Denmark [4, 5]. Our analysis indicates that recent estimates of the European CKD Burden Consortium probably overestimated the German CKD prevalence, and a comparison with data from 2008 to 2011 suggest stable numbers over the past decade for the investigated age group [5, 6]. Indeed, the global age-adjusted CKD prevalence also remained stable, and increases in cases are mainly attributed to the aging population [2]. Four in five individuals with an at least moderately reduced eGFR had a uACR <30 mg/g. This is of pivotal importance, as nearly all CKD treatments, such as inhibitors of the renin–angiotensin–aldosterone system or the sodium glucose transporter 2, only have proven benefit in individuals with proteinuria [7, 8]. Thus, there are still few proven therapeutic options for most individuals with CKD, highlighting the need for further research on therapies for non-proteinuric CKD.

High numbers of CKD were found not only in individuals with hypertension or diabetes, but also in males. This is in contrast to the global distribution of CKD which shows higher rates in females [2]. However, the association of male sex with CKD observed in our study was partially attributable to confounding comorbid conditions such as diabetes and hypertension. Thus, differences in the sex-specific CKD prevalence between studies might also underly geographic or genetic variations in risk factors. Still, the observed predominance of males with CKD cuold have important implications for healthcare strategies, as intensified screening in urban European populations such as Hamburg may be specifically beneficial for males.

The impact of different eGFR formulas on the CKD prevalence was substantial. CKD prevalence increased by 8% using the 2012 CKD-EPI formula for cystatin C as compared with the 2009 CKD-EPI formula for creatinine. Such differences can implicate important medical and socioeconomic consequences with substantial consequences for healthcare expenses. Though cystatin C can outperform creatinine in cardiovascular risk prediction, future studies will have to clarify whether the broad use of cystatin C improves medical care, reduces outcomes and is cost-effective [9].

Although this study constitutes one of the largest on the CKD prevalence and characteristics in Germany and closes the gap of missing current data, it faces limitations. These include a possible selection bias including a potential selection of healthier individuals leading to an underestimation of CKD, lack of information on non-participants, a low proportion of non-Caucasians, missing GFR measurements with exogenous markers and no validation of self-reported information. As a cross-sectional study, repeated kidney function measurements to confirm CKD are missing. Also, the study included an urban German population, and its validity in other populations remains unknown.

In conclusion, this study shows a high CKD prevalence in a large population-based German cohort. Besides correlations with traditional risk factors, CKD shares notable sex differences with higher rates in males, partially attributed to an increased comorbidity burden in male individuals. These findings can help in guiding CKD screening strategies.

## Supplementary Material

gfaf075_Supplemental_File

## Data Availability

Data will be shared upon reasonable request to the corresponding author. Data sharing is subject to the approval of the steering committee of the Hamburg City Health Study.
