# Supplementary material for: Prevalence and characteristics of chronic kidney disease in the Hamburg City Health Study
Source: Nephrol Dial Transplant. 2025 Apr 30;40(8):1632–4. doi: 10.1093/ndt/gfaf075 (PMC12315801; doi:10.1093/ndt/gfaf075)
Supplement: gfaf075_Supplemental_File [file gfaf075_supplemental_file.docx]

**Supplementary Material**

**Item S1.** Supplementary methods.

**Figure S1.** eGFR and uACR distribution in the HCHS.

**Table S1.** Prevalence of CKD and eGFR stages according to different GFR estimations.

**Table S2.** Comparison of dipstick proteinuria to uACR.

**Table S3.** Prevalence of CKD in subgroups.

**Table S4.** Association of selected risk factors with CKD prevalence.

**Table S5.** Distribution of selected risk factors by sex.

**Table S6.** Number of missing values.

**Table S7.** Sensitivity analysis on unimputed dataset.

**Item S1. Supplementary methods.**

**Study design and population**

The design and protocol of the HCHS have been published.^1^ Briefly, the HCHS is an ongoing and prospective population-based cohort study aiming to investigate prognostic factors for major chronic disease. It encompasses inhabitants of the city of Hamburg, Germany, aged 45 to 74 years. Participants are randomly selected from the official resident registry and invited to participate in the study via mail. This mail includes an invitation letter and an informational leaflet outlining key de-tails about the study. Appointments are scheduled by participants themselves at the epidemiological study center of the University Medical Center Hamburg-Eppendorf. After providing written informed consent, all participants undergo a detailed assessment of various organ functions, medical history, vital status, and demographics including medical questionnaires, specific examinations, laboratory analyses, and establishment of a biobank. The study adheres to the principles of the Declaration of Helsinki and is registered at ClinicalTrials.gov (NCT03934957). The current analysis provides a cross-sectional investigation of kidney parameters within the first 10000 participants, enrolled between February 2016 and November 2018. Individuals after kidney transplantation (15 participants) or receiving chronic dialysis treatment (2 participants) were excluded.

**Assessment of kidney function within the HCHS**

Data on kidney function investigated in the HCHS include questionnaires, renal ultrasound as well as blood and urine analyses. Questionnaires comprise information on symptoms such as bloody or foamy urine, drinking and urinating habits, a family history of kidney diseases, information on prior kidney replacement therapies or kidney transplantation, and awareness of a diagnosed kidney disease including its nature (kidney diseases are categorized into diabetic kidney disease, hypertensive kidney disease, glomerulonephritis, autosomal dominant polycystic kidney disease, other, or unknown).

Laboratory analyses are performed according to standardized protocols with the laboratory personal being blinded to the participants’ information.^2^ Creatinine is measured directly at the study visit using Atellica CH Creatinine_2 assays with a measurement range of 0.15 - 30 mg/dl (Siemens Healthineers, Erlangen, Germany). Cystatin C measurements are performed with Abbott MULTIGENT assays having a measurement range of 0.05 - 9.62 mg/l on frozen samples stored at -80 °C. Urine analyses are performed on a first-morning voided spot urine. An automated dipstick investigation is conducted during study visits with Multistix 10 SG reagent stripes on a CLINITEK Status+ analyzer (Siemens Healthineers, Erlangen, Germany). Quantification of albumin (measurement range 3 - 380 mg/l; in dilution up to 3800 mg/l; Atellica CH Microalbumin assay), total protein (10 - 1250 mg/l; in dilution up to 5000 mg/l; Atellica CH Total Protein assay), creatinine (3.0 - 245 mg/dl; in dilution up to 735 mg/dl; Atellica CH Creatinine), sodium (2 - 300 mmol/l; in dilution up to 600 mmol/l), and potassium (2 - 300 mmol/l; in dilution up to 600 mmol/l; A-LYTE Integrated Multisensor assay) are performed with an Atellica CH analyzer (Siemens Healthineers, Erlangen, Germany) on urine samples stored at -80 °C. The uACR as well as the urinary protein-to-creatinine ratio (uPCR) are calculated with these results. Comparison of uPCR and urine dipstick proteinuria to uACR categories is performed using the 2024 Kidney Disease: Improving Global Outcomes (KDIGO) recommendations for conversion.^3^

**Estimation of GFR and CKD staging**

For the current analysis we estimated the GFR with the 2009 Chronic Kidney Disease Epidemiology Collaboration (CKD-Epi) formula for creatinine, referred to as CKD-Epi_Creat_, since this currently is the most widely used formula in clinical practice.^4^ Secondary analyses were performed using the 2012 CKD-Epi formula for cystatin C (CKD-Epi_Cys_), the 2012 CKD-Epi formula for the combination of creatinine and cystatin C (CKD-Epi_Creat/Cys_),^5^ the race-free CKD-Epi formula for creatinine (CKD-Epi-RF_Creat_) or the combination of creatinine and cystatin C (CKD-Epi-RF_Creat/Cys_)^6^ as well as the European Kidney Function Consortium (EKFC) equation for creatinine (EKFC_Creat_), cystatin (EKFC_Cys_) or the combination of both (EKFC_Creat/Cys_).^7,8^ CKD was diagnosed by an eGFR < 60 ml/min/1.73m^2^ or an uACR ≥ 30 mg/g and staged according to the KDIGO criteria.^3^

**Assessment of non-kidney data**

Vital status was assessed using standardized procedures.^9^ Data on socioeconomics, smoking status (never, former, or current), physical activity (hours of physical activity per week), medication, as well as the diagnoses of peripheral artery disease, coronary heart disease, and malignancies derive from standardized patient interviews. Education level was classified according to the International Standard Classification of Education.^10^ We based the definition of other comorbidities on composite criteria. Hypertension was defined as a systolic blood pressure ≥ 140 mmHg, a diastolic blood pressure ≥ 90 mmHg, treatment with antihypertensive medication, or a self-reported diagnosis of hypertension. Definition of diabetes comprised a fasting blood glucose level ≥ 126 mg/dL, a non-fasting blood glucose ≥ 200 mg/dL, a HbA1c ≥ 6.5, treatment with antidiabetic drugs, or a self-reported diagnosis of diabetes. Atrial fibrillation was based on a self-reported diagnosis or evidence on an electrocardiogram obtained during study visit. Treatment with lipid lowering drugs or a ratio of low to high density lipoproteins > 3.5 was used to assess dyslipidemia. Heart failure was defined according to the 2021 European Society of Cardiology (ESC) guidelines using clinical, echocardiographic and laboratory information.^11^ All reported non-kidney laboratory tests were conducted at the study visit on an Atellica COAG 360 System analyzer (Siemens Healthineers, Erlangen, Germany).

**Statistical analyses**

Continuous variables are presented as median and interquartile ranges (IQR), categorical variables as numbers and percentages. For prevalence data, 95% confidence intervals (CI) derive from Wilson score intervals. Distribution of the eGFR and uACR as well as the CKD prevalence are reported for the total study population as well as for subgroups stratified by age, sex, hypertension, or diabetes. Individual differences of the various eGFR formulas to the commonly used CKD-Epi_Creat_ formula are presented and compared using paired t-test. Also, we analyzed the independent association of potential demographic and comorbid risk factors with the prevalence of CKD in uni- and multivariable logistic or linear regression models. Potential risk factors were selected based on current literature and pathophysiological knowledge and included age, sex, body mass index (BMI), hypertension, diabetes, dyslipidemia, and sport activity. Continuous variables with skewed distribution were logarithmized before analyzed in regression models (sport activity). Results of logistic and linear regression models are reported as odds ratio and effect estimate with 95%-CI, respectively. Missing values were imputed with random forest-based imputations (see supplementary Table S6 for the proportion of missing values and Table S7 for a sensitivity analysis of the primary outcome on the unimputed dataset).^12^ Analyses were performed using R version 4.1.1 (R Foundation for Statistical Computing, Vienna, Austria).

**References**

1. Jagodzinski A, Johansen C, Koch-Gromus U, et al. Rationale and Design of the Hamburg City Health Study. Eur J Epidemiol 2020;35(2):169-181. (In eng). DOI: 10.1007/s10654-019-00577-4.

2. Schmidt-Lauber C, Hanzelmann S, Schunk S, et al. Kidney outcome after mild to moderate COVID-19. Nephrol Dial Transplant 2023;38(9):2031-2040. DOI: 10.1093/ndt/gfad008.

3. Kidney Disease: Improving Global Outcomes CKDWG. KDIGO 2024 Clinical Practice Guideline for the Evaluation and Management of Chronic Kidney Disease. Kidney Int 2024;105(4S):S117-S314. DOI: 10.1016/j.kint.2023.10.018.

4. Levey AS, Stevens LA, Schmid CH, et al. A new equation to estimate glomerular filtration rate. Ann Intern Med 2009;150(9):604-612. (10.7326/0003-4819-150-9-200905050-00006). DOI: papers3://publication/doi/10.7326/0003-4819-150-9-200905050-00006.

5. Inker LA, Schmid CH, Tighiouart H, et al. Estimating glomerular filtration rate from serum creatinine and cystatin C. N Engl J Med 2012;367(1):20-9. DOI: 10.1056/NEJMoa1114248.

6. Inker LA, Eneanya ND, Coresh J, et al. New Creatinine- and Cystatin C-Based Equations to Estimate GFR without Race. N Engl J Med 2021;385(19):1737-1749. DOI: 10.1056/NEJMoa2102953.

7. Pottel H, Björk J, Courbebaisse M, et al. Development and Validation of a Modified Full Age Spectrum Creatinine-Based Equation to Estimate Glomerular Filtration Rate : A Cross-sectional Analysis of Pooled Data. Annals of internal medicine 2021;174(2):183-191. (In eng). DOI: 10.7326/m20-4366.

8. Pottel H, Bjork J, Rule AD, et al. Cystatin C-Based Equation to Estimate GFR without the Inclusion of Race and Sex. N Engl J Med 2023;388(4):333-343. DOI: 10.1056/NEJMoa2203769.

9. Schmidt-Lauber C, Alba Schmidt E, Hänzelmann S, et al. Increased blood pressure after nonsevere COVID-19. J Hypertens 2023;41(11):1721-1729. (In eng). DOI: 10.1097/hjh.0000000000003522.

10. OECD. Manual for ISCED-97 Implementation in OECD Countries. Paris, 1999.

11. Wenzel JP, Nikorowitsch J, Bei der Kellen R, et al. Heart failure in the general population and impact of the 2021 European Society of Cardiology Heart Failure Guidelines. ESC Heart Fail 2022;9(4):2157-2169. DOI: 10.1002/ehf2.13948.

12. Stekhoven DJ, Bühlmann P. MissForest--non-parametric missing value imputation for mixed-type data. Bioinformatics 2012;28(1):112-8. (In eng). DOI: 10.1093/bioinformatics/btr597.

**Figure S1. eGFR and uACR distribution in the HCHS.**

Illustration of eGFR (a) and uACR (b) within the study population. The vertical orange line in panel (a) indicates an eGFR < 60 ml/min/1.73m^2^ and the insert in panel (b) shows the uACR distribution in greater detail. eGFR: estimated glomerular filtration rate; HCHS: Hamburg City Health Study; uACR: urinary albumin-to-creatinine ratio.

**Table S2. Prevalence of CKD and eGFR stages according to different GFR estimations.**

|  | **Creatinine** | | | **Cystatin C** | | **Creatinine/cystatin C** | | |
| --- | --- | --- | --- | --- | --- | --- | --- | --- |
|  | **CKD-Epi_Creat_** | **CKD-Epi-RF_Creat_** | **EKFC_Creat_** | **CKD-Epi_Cys_** | **EKFC_Cys_** | **CKD-Epi_Creat/Cys_** | **CKD-Epi-RF_Creat/Cys_** | **EKFC_Creat/Cys_** |
| **CKD**  **(eGFR ≤ 60 ml/min/1.73 m^2^**  **or uACR ≥ 30 mg/g)** | 1 115 (11.2) | 919 (9.2) | 1 400 (14) | 1 945 (19.5) | 1 630 (16.3) | 1 315 (13.2) | 1 100 (11.1) | 1 331 (13.1) |
| **eGFR stages (ml/min/1.73 m^2^)** |  |  |  |  |  |  |  |  |
| ≥ 90 | 3 714 (37.2) | 5 116 (51.2) | 2 146 (21.5) | 2 017 (20.2) | 1 430 (14.3) | 2 575 (25.8) | 3 752 (25.9) | 1 503 (15.1) |
| < 90 - 60 | 5 670 (56.8) | 4 478 (44.9) | 6 909 (69.2) | 6 409 (64.2) | 7 335 (73.5) | 6 553 (65.6) | 5 628 (56.4) | 7 602 (76.1) |
| < 60 | 599 (6) | 389 (3.9) | 928 (9.3) | 1 557 (15.6) | 1 218 (12.2) | 855 (8.6) | 613 (6.1) | 878 (8.8) |
| < 60 - 30 | 584 (5.8) | 376 (3.8) | 909 (9.1) | 1 511 (15.4) | 1 192 (11.9) | 827 (8.3) | 583 (5.8) | 864 (8.7) |
| < 30 | 15 (0.2) | 13 (0.1) | 19 (0.2) | 46 (0.5) | 26 (0.3) | 28 (0.3) | 30 (0.3) | 14 (0.1) |

Numbers are counts with percentages in brackets. Percentages do not sum up to 100 % because of rounding. CKD: chronic kidney disease; eGFR: estimated glomerular filtration rate, uACR: urinary albumin-to-creatinine ratio.

**Table S3. Comparison of dipstick proteinuria to uACR.**

|  | **urine dipstick proteinuria** | | | | |
| --- | --- | --- | --- | --- | --- |
| **uACR** | **negative**  (n = 9 428) | **trace**  (n = 206) | **+ [~30 mg/g]**  (n = 227) | **++ [~100 mg/g]**  (n = 105) | **+++ [≥ 300 mg/g]**  (n = 17) |
| **< 30 mg/g**  (n = 9 351) | 9 112 (91.3) | 150 (15.0) | 82 (0.8) | 6 (0.1) | 1 (0) |
| **30 - < 300 mg/g**  (n = 559) | 307 (3.1) | 56 (0.6) | 134 (1.3) | 61 (0.6) | 1 (0) |
| **≥ 300 mg/g**  (n = 73) | 9 (0.9) | 0 | 11 (0.1) | 38 (0.4) | 15 (0.2) |

Numbers are counts with percentages in brackets. Percentages do not sum up to 100 % because of rounding. Corresponding categories according to the Kidney Disease: Improving Global Outcomes (KDIGO) recommendations are marked in grey. uACR: urinary albumin-to-creatinine ratio.

**Table S4.** **Prevalence of CKD in subgroups.**

| **Subgroup** | **eGFR** | | **uACR** | | **CKD** | **P-value** |
| --- | --- | --- | --- | --- | --- | --- |
|  | ml/min/1.73 m^2^  median (IQR) | < 60 ml/min/1.73 m^2^  n (%) | mg/g  median (IQR) | > 30 mg/g  n (%) | n (%) |  |
| **Age** |  |  |  |  |  |  |
| 45-54 *(n = 2 270)* | 95 (86; 102) | 18 (0.8) | 4 (3; 7) | 68 (3) | 83 (3.7) | Ref. |
| 55-64 *(n = 3 317)* | 88 (79; 96) | 94 (2.8) | 4 (3; 7) | 150 (4.5) | 229 (6.9) | < 0.001 |
| 65-74 *(n = 4 396)* | 79 (68; 88) | 487 (11.1) | 6 (3; 11) | 414 (9.4) | 803 (18.3) | < 0.001 |
| **Sex** |  |  |  |  |  |  |
| Male *(n = 4 884)* | 85.8 (75.4; 94) | 332 (6.8) | 4 (2; 9) | 390 (8) | 640 (13.1) | < 0.001 |
| Female *(n = 5 099)* | 85 (75; 94) | 267 (5.2) | 5 (3; 9) | 242 (4.7) | 475 (9.3) |  |
| **Hypertension** |  |  |  |  |  |  |
| No *(n = 3 518)* | 90 (80; 97) | 69 (2) | 4 (2; 6) | 95 (2.7) | 159 (4.5) | < 0.001 |
| Yes *(n = 6 465)* | 83 (72; 92) | 530 (8.2) | 5 (3; 10) | 537 (8.3) | 956 (14.8) |  |
| **Diabetes** |  |  |  |  |  |  |
| No *(n = 9 179)* | 86 (76; 94) | 465 (5.1) | 5 (3; 8) | 475 (5.2) | 872 (9.5) | < 0.001 |
| Yes *(n = 804)* | 79 (66; 89) | 134 (16.7) | 8 (4; 21) | 157 (19.5) | 243 (30.2) |  |

CKD was defined as an eGFR < 60 ml/min/1.73m^2^ or an uACR ≥ 30 mg/g. CKD: chronic kidney disease; eGFR: estimated glomerular filtration rate; uACR: urinary albumin-to-creatinine ratio. Chi-squared test was used to test for differences in CKD prevalence between groups.

**Table S1. Distribution of selected risk factors by sex.**

| **Characteristics** | **Females**  **(n = 5099)** | **Males**  **(n = 4884)** |
| --- | --- | --- |
| Age in years (median (IQR)) | 62 (55; 69) | 64 (56; 70) |
| BMI in kg/m² (median (IQR)) | 25.4 (22.8; 28.7) | 26.7 (24.6; 29.4) |
| Dyslipidemia (%) | 854 (16.7) | 1419 (29.1) |
| Diabetes (%) | 304 (6.0) | 500 (10.2) |
| Hypertension (%) | 2969 (58.2) | 3496 (71.6) |
| Education |  |  |
| High | 1765 (34.6) | 2690 (55.1) |
| Medium | 3027 (59.4) | 2036 (41.7) |
| Low | 307 (6.0) | 158 (3.2) |
| Physical activity in hours per week (median (IQR)) | 2 (0.8; 3.5) | 2 (0; 4) |

BMI: body mass index, IQR: interquartile range.

**Table S5. Association of selected risk factors with CKD prevalence.**

|  | **Unadjusted model** | |  | | **Adjusted model** | | |  |
| --- | --- | --- | --- | --- | --- | --- | --- | --- |
|  | *Odds ratio (95%-CI)* | *P-value* | |  | | *Odds ratio (95%-CI)* | *P-value* | |
| Age in years | 1.01 (1.01; 1.01) | <0.001 | |  | | 1.01 (1.01; 1.01) | < 0.001 | |
| Female sex | 0.96 (0.95; 0.97) | < 0.001 | |  | | 0.99 (0.98; 1) | 0.045 | |
| BMI in kg/m² | 1.01 (1.01; 1.01) | < 0.001 | |  | | 1 (1; 1.01) | < 0.001 | |
| Education |  |  | |  | |  |  | |
| Low | Ref. |  | |  | | Ref. |  | |
| Medium | 0.96 (0.93; 0.99) | 0.019 | |  | | 0.99 (0.96; 1.02) | 0.567 | |
| High | 0.94 (0.91; 0.97) | < 0.001 | |  | | 1.00 (0.98; 1.01) | 0.418 | |
| Log(sport in hours per week) | 1 (1; 1) | < 0.001 | |  | | 1 (1; 1) | 0.014 | |
| Hypertension | 1.11 (1.09; 1.13) | < 0.001 | |  | | 1.03 (1.02; 1.05) | < 0.001 | |
| Diabetes | 1.23 (1.20; 1.26) | < 0.001 | |  | | 1.15 (1.12, 1.18) | < 0.001 | |
| Dyslipidemia | 1.12 (1.09; 1.13) | < 0.001 | |  | | 1.04 (1.03; 1.06) | < 0.001 | |

Logistic regression models showing the association of pre-defined potential risk factors with the prevalence of CKD. CKD was defined as an eGFR < 60 ml/min/1.73m^2^ or an uACR ≥ 30 mg/g. Adjustment was performed for all listed potential risk factors. BMI: body mass index; eGFR: estimated glomerular filtration rate; uACR: urinary albumin-to-creatinine ratio.

**Table S6. Number of missing values.**

| **Characteristics, n (%)** | **Missing values** |
| --- | --- |
| Age | 0 (0) |
| Sex | 0 (0) |
| BMI | 559 (6) |
| Heart rate | 409 (4) |
| Systolic blood pressure | 409 (4) |
| Diastolic blood pressure | 409 (4) |
| Ethnicity | 30 (0) |
| Hypertension | 469 (5) |
| Dyslipidemia | 709 (7) |
| Malignancies | 759 (8) |
| Diabetes | 789 (8) |
| Coronary heart disease | 829 (8) |
| Heart failure | 2 096 (21) |
| Peripheral artery disease | 849 (9) |
| Employment | 1 168 (12) |
| Education | 579 (6) |
| Smoking status | 50 (1) |
| Sports | 1 338 (13) |
| Medication | 469 (5) |
| Hemoglobin | 309 (3) |
| HBA1c | 369 (4) |
| Sodium | 299 (3) |
| Potassium | 299 (3) |
| Creatinine | 319 (3) |
| Cystatin C | 918 (9) |
| uACR | 1 587 (16) |
| uPCR | 1 597 (16) |

**Table S7. Sensitivity analysis on unimputed dataset.**

| **Population** | **eGFR** | | **uACR** | | **CKD** |
| --- | --- | --- | --- | --- | --- |
|  | ml/min/1.73 m^2^  median (IQR) | < 60 ml/min/1.73 m^2^  n (%) | mg/g  median (IQR) | > 30 mg/g  n (%) | n (%) |
| **Overall** | 86 (75; 94) | 582 / 9 664 (6) | 4 (3; 9) | 594 /8 397 (7.1) | 1 087 / 9 983 (10.9) |
| **Sex** |  |  |  |  |  |
| Male | 86 (75; 94) | 321 / 4 750 (6.8) | 4 (2; 8) | 362 / 4 112 (8.8) | 621 / 4 884 (12.7) |
| Female | 86 (75; 94) | 261 / 4 888 (5.3) | 5 (3; 9) | 232 / 4 285 (5.4) | 466 / 5 099 (9.1) |

CKD was defined as an eGFR < 60 ml/min/1.73m^2^ or an uACR ≥ 30 mg/g. CKD: chronic kidney disease; eGFR: estimated glomerular filtration rate; uACR: urinary albumin-to-creatinine ratio.
